# Propranolol-Loaded Trehalosome as Antiproliferative Agent for Treating Skin Cancer: Optimization, Cytotoxicity, and In Silico Studies

**DOI:** 10.3390/pharmaceutics15082033

**Published:** 2023-07-28

**Authors:** Mona K. Younis, Yara E. Elakkad, Rasha R. Fakhr Eldeen, Isra H. Ali, Islam A. Khalil

**Affiliations:** 1 Department of Pharmaceutics, College of Pharmacy and Drug Manufacturing, Misr University of Science and Technology, 6th of October City 12566, Egypt; mona.younis@must.edu.eg (M.K.Y.); yara.mohamed@must.edu.eg (Y.E.E.); 2 Department of Biochemistry, College of Pharmacy and Drug Manufacturing, Misr University of Science and Technology, 6th of October City 12566, Egypt; rasha.rashid@must.edu.eg; 3 Department of Pharmaceutics, Faculty of Pharmacy, University of Sadat City, Sadat City 32897, Egypt; isra.ali@fop.usc.edu.eg

**Keywords:** propranolol, trehalosomes, drug repurposing, skin cancer

## Abstract

This study aims at preparing propranolol-loaded trehalosomes (a trehalose-coated liposome) to be used as an antiproliferative agent for treating skin cancer. A factorial design was used to select the optimum formula, where trehalose, lecithin, and Tween 80 levels were studied. A total of 24 runs were prepared and characterized according to size, charge, entrapment efficiency, and release after 3 h to select the optimum formula. The optimized formula was investigated using TEM, DSC, and FTIR. Cell studies were carried out against the human melanoma cell line to measure cytotoxicity, apoptosis/necrosis, and cell cycle arrest. In silico studies were conducted to understand the interaction between propranolol and the influential receptors in melanoma. The results showed the selected formula consisted of trehalose (175 mg), lecithin (164 mg), and Tween 80 (200 mg) with a size of 245 nm, a charge of −9 mV, an EE% of 68%, and a Q3 of 62%. Moreover, the selected formula has good cytotoxicity compared to the free drug due to the synergistic effect of the drug and the designed carrier. IC_50_ of free propranolol and the encapsulation of propranolol were 17.48 μg/mL and 7.26 μg/mL, respectively. Also, propranolol and the encapsulation of propranolol were found to significantly increase early and late apoptosis, in addition to inducing G1 phase cell cycle arrest. An in silico virtual study demonstrated that the highest influential receptors in melanoma were the vitamin D receptor, CRH-R1, VEGFR 1, and c-Kit, which matches the results of experimental apoptotic and cell cycle analysis. In conclusion, the selected formula has good cytotoxicity compared to the free drug due to the synergistic effect of the drug and the designed carrier, which make it a good candidate as an antiproliferative agent for treating skin cancer.

## 1. Introduction

One of the biggest challenges in cancer research is understanding how cancer immune-tolerance mechanisms work. Melanoma is a highly aggressive tumor with low five-year survival rates. It has been discovered that β2-adrenoceptors play a role in cancer and are a new target in treating melanoma. Recent studies have shown that β3-adrenoceptors have a pleiotropic effect on the melanoma microenvironment, leading to cancer progression, but the mechanisms are still not well-understood. Beta-blockers are widely used therapeutic agents and have shown potential anticarcinogenic effects in patients with prostate cancer, breast cancer, and melanoma. However, it should also be noted that β-blockers can cause or aggravate various skin conditions such as psoriasis, lichen planus-like drug eruptions (LDE), acrocyanosis, and alopecia [1].

Propranolol (PPL) is a non-selective beta-blocker that has evidence supporting its use in the long-term management of chronic stable angina and as an antihypertensive. It is also highly effective in preventing and reducing certain types of migraine headaches [2]. Recent studies have been presented to repurpose propranolol as an anticancer agent [3,4]. PPL has been extensively studied, and its tolerability, low cost, and efficacy in vitro in preventing tumor progression make it a promising candidate for repurposing in cancer treatment for humans [3]. Although there are limited published prospective human clinical studies, current trials are underway investigating the use of ß-blockers in cancer. Two recently published studies evaluated the use of propranolol in the peri-operative period, with one evaluating the use of a consistently lower dose of propranolol along with a cyclooxygenase-2 inhibitor in breast cancer patients, while another study assessed the use of propranolol in ovarian cancer patients [2,4]. A third non-randomized study showed that propranolol protects patients with thick cutaneous melanoma from disease recurrence [5,6].

The first-line treatment for cancer typically involves surgical removal of solid tumors, radiation therapy, and chemotherapy. However, the systemic administration of free drugs is considered a major clinical failure of chemotherapy as a limited drug concentration reaches the tumor site. Most active pharmaceutical ingredients used in chemotherapy are highly cytotoxic to both cancer and normal cells, making it essential to target tumor vasculatures. Encapsulating anti-cancer drugs within the liposomal system offers a secure platform for the targeted delivery of anti-cancer drugs, reducing cytotoxic side effects on normal cells. Therefore, the current study focuses on the loading of PPL into a transdermal lipid vesicular system, which will have several advantages, such as improved patient compliance in long-term therapy, bypassing first-pass metabolism, increasing drug bioavailability, delaying the elimination of highly metabolized drugs, extending drug circulation life, targeting drug delivery, as well as improving drug stability [7,8].

Trehalose (α-d-glucopyranosyl-(1→1)-α-d-glucopyranoside) is a naturally occurring alpha-linked disaccharide comprised of two molecules of glucose [9]. Studies reported that trehalose in a concentration of 5% inhibits the proliferation of melanoma cells [10]. It can also protect important cellular components, where it can form special structures over the cell membrane that can protect molecular structures from damage or destruction [11]. Also, trehalose was used as a coating material for nanocomplexes to promote stabilization and effective polyplex-mediated siRNA delivery [12]. Moreover, trehalosomes were used as colon-targeting trehalose-based green nanocarriers for the maintenance of remission in inflammatory bowel diseases [13]. Therefore, trehalose was used in the current study to decorate the liposomes due to its anti-proliferation activity against melanoma cells, in addition to serving as a stabilizer for the vesicle structure.

**Scheme 1:**
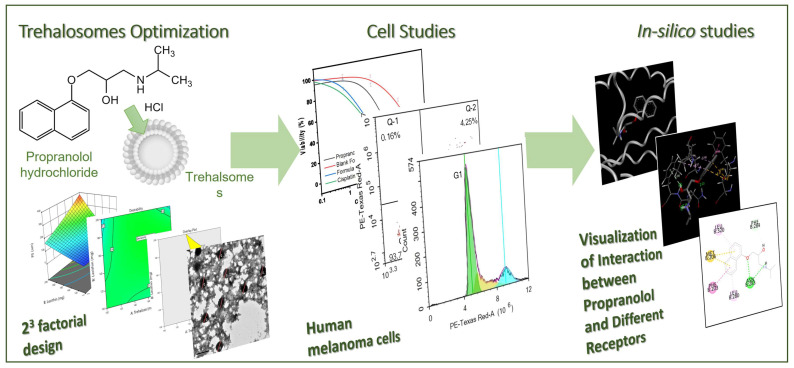
Schematic diagram for the workflow of the current study.

In the current study (Scheme 1), PPL was loaded with trehalosomes (PTHs) using the ethanol injection method. Different factors were studied to optimize the formula using 2^3^ factorial design (trehalose, lecithin, and tween 80 weight). To select the optimum formula, different responses were measured (size, charge, entrapment efficiency, and release after 3 h). Afterward, the optimum formula was characterized by its physicochemical properties. In vitro cell line studies were conducted for cytotoxicity, necrosis and apoptosis assay, and cell cycle analysis. Moreover, in silico studies were conducted to understand the mechanism of cytotoxicity.

## 2. Materials and Methods

### 2.1. Materials

Propranolol hydrochloride was purchased from Sigma-Aldrich (Saint Louis, MO, USA). Trehalose was purchased from Oxford Lab fine chem LLP (Mumbai, India). Lecithin from soyabean and Tween 80 were purchased from Alpha Chemika Company (Mumbai, India). Potassium dihydrogen phosphate and disodium hydrogen phosphate were supplied from El Nasr Pharmaceutical Chemicals (Cairo, Egypt). DMEM media, streptomycin, penicillin, heat-inactivated fetal bovine serum (FBS), and DNAse/RNAse-free water were purchased from Thermo Fisher Scientific (Cleveland, OH, USA).

### 2.2. Preparation of PPL-Loaded Trehalosomes (PTHs)

PPL (10 mg), lecithin, and Tween 80 were dissolved in ethanol using a magnetic stirrer at 60 °C. Trehalose was dissolved in distilled water. A dropwise addition of the organic phase to the aqueous phase was conducted with the aid of a stirrer for 30 min at 60 °C, then left at room temperature to cool. PTHs dispersions were sonicated for 2 min (5 s on/off; Ultrasonic LC 60 H, Elma, Singem, Germany). The prepared PTHs were kept in a refrigerator for further investigation [13].

### 2.3. Experimental Design

An experimental design was conducted with the aid of Design-Expert^®^ Software version 13 (Stat-Ease, Inc., Minneapolis, MN, USA) using 2^3^ factorial design. In the study, the independent variables were trehalose (X1), lecithin (X2), and Tween 80 (X3). PTHs were prepared formulations as demonstrated in Table 1.

Each formulation was prepared 3 times according to the levels demonstrated in Table 2 to calculate the mean and standard deviation. The prepared formulations were characterized, where the dependent variables were particle size, charge, entrapment efficiency percent, and percentage of drug released after 3 h, as shown in Table 3. The optimized formula was selected according to the lowest size and the highest charge (absolute value), EE %, and 3 h dissolution, as shown in Table 3.

### 2.4. Characterization of Prepared Trehalosomes

#### 2.4.1. Particle Size and Surface Charge

The particle size and surface charge of the prepared PTHs were determined using Malvern Nano-ZS (Malvern Instruments Ltd., Worcestershire, UK) after appropriate dilution with distilled water at a temperature of 25 °C [14].

#### 2.4.2. Entrapment Efficiency (EE)

Entrapment efficiency (EE) was determined by indirect technique, where PTHs were separated from the dispersion by an ultracentrifugation method at 15,000 rpm for 1 h at 4 °C. The collected pellets were washed 3 times and added to the supernatant. Finally, the PPL concentration was measured spectrophotometrically (Shimadzu UV-1600, Kyoto, Japan) in the supernatant at a wavelength of 290 nm. The entrapment efficiency was calculated according to the following equation:

(1) $$\mathrm{EE}\%=\left(\mathrm{Total}\;\mathrm{PPL}-\mathrm{Unentraped}\;\mathrm{PPL}\right)/\left(\mathrm{Total}\;\mathrm{PPL}\right)\times 100$$

#### 2.4.3. In Vitro Release Studies

The dissolution profile of PPL was determined using the dissolution apparatus (Dr. Schleuniger Pharmaton, type Diss 6000, Solothurn, Switzerland). An amount of 1 mL of PTH was added to a dialysis tube (12 kDa, Sigma-Aldrich, USA), attached to a dissolution paddle, and then placed into a dissolution vessel containing 100 mL phosphate buffer. The solubility was tested prior to the test to confirm the maintenance of the sink condition. The release condition was stirred at 100 rpm and the temperature was maintained at 37 °C. Samples were collected each hour and replaced by fresh buffer. The concentration of the released PPL was measured by spectrophotometer at 290 nm and the cumulative release profile was determined.

#### 2.4.4. Transmission Electron Microscopy (TEM)

The morphological examination of the optimum PTHs was studied by transmission electron microscopy (Joel JEM 1230, Tokyo, Japan). The PTH sample was dried on a carbon-coated grid and stained with phosphotungstic acid (2%). After the drying of the phosphotungstic acid, the images were acquired using TEM at 200 kV.

#### 2.4.5. Differential Scanning Calorimetry (DSC)

DSC thermograms of PPL, lecithin, trehalose, Tween 80, and the selected formula were generated by differential scanning calorimetry equipment (DSC50, Shimadzu, Japan) connected to TA-501 thermal analyzer. A total of 5 mg of each sample was heated in a sealed aluminum pan at a temperature range of 25 to 300 °C at a heating rate of 10 °C/min, under a nitrogen flow of 20 mL/min.

#### 2.4.6. Fourier-Transform Infrared Spectroscopy (FTIR)

FTIR spectra of PPL, lecithin, trehalose, Tween 80, and the selected formula were obtained by FTIR spectrophotometer (FTIR-8400, Shimadzu, Japan). Then, 3 mg of each sample was mixed with KBr and compressed into a disk, followed by scanning at a range from 400 to 4000 cm^−1^.

### 2.5. In Vitro Cell Study

#### 2.5.1. Cytotoxicity Cell Study

The cell viability was measured using Sulforhodamine B (SRB) assay. Human melanoma cells A375 (ATCC^®^ CRL-1619™) were used to test unloaded and loaded formula, PPL, and cisplatin as control. Aliquots of 100 μL cell suspension (5 × 103 cells) were incubated in complete media for 24 h. The cells were treated with 100 μL media containing different samples at various concentrations (five concentrations prepad in cells media were used). After 72 h of drug exposure, the cells were fixed by replacing media with 150 μL of 10% Trichloroacetic acid (TCA) and incubated at 4 °C for 1 h. The TCA was removed, and the cells were washed 5 times with distilled water. An amount of 70 μL of 0.4% *w*/*v* SRB solution was mixed and incubated in a dark place at room temperature for 10 min, followed by washing 3 times with 1% acetic acid and drying overnight. Then, 150 μL of 10 mM of Tris base solution (TRIS) was added to dissolve the protein-bound SRB stain. The absorbance was measured at 540 nm using a BMG LABTECH^®^-FLUOstar Omega microplate reader (Ortenberg, Germany) [15]. The cytotoxicity was expressed as a Half-maximal inhibitory concentration (IC50). The IC50 was determined by plotting dose–response curves, where IC50 values were obtained from dose–response curves and regression analysis by GraphPad PRISM version 6 program (GraphPad Software, Inc., San Diego, CA, USA).

#### 2.5.2. Flow Cytometric Analysis

Apoptosis/necrosis and cell cycle distribution were investigated by flow cytometric analysis (ACEA Biosciences Inc., San Diego, CA, USA) as reported [15]. Human melanoma cells (A375) were maintained in DMEM media, then 100 mg/mL of streptomycin, 100 units/mL of penicillin, and 10% of heat-inactivated fetal bovine serum in a humidified, 5% (*v*/*v*) carbon dioxide atmosphere were added at 37 °C.

##### Annexin-V-FITC Apoptosis Assay

Apoptotic cells were determined using an Annexin V-FITC apoptosis detection kit (Abcam Inc., Cambridge Science Park, Cambridge, UK). Cells were treated with different samples for 48 h. According to the manufacturer protocol, 105 cells were collected by trypsinization, washed 2 times with ice-cold PBS (pH 7.4), and incubated in the dark with 0.5 mL of Annexin V-FITC/PI solution for 30 min at room temperature. After staining, the cells were injected via a flow cytometer and analyzed for fluorescent signals FITC (FL1, λex/em 488/530 nm) and PI (FL2, λex/em 535/617 nm). For each sample, 12,000 events were acquired and positive FITC and/or PI cells were quantified by quadrant analysis and calculated using ACEA NovoExpress™ software version 1.3 (ACEA Biosciences Inc., San Diego, CA, USA) [15].

##### Cell Cycle Analysis

The cell distribution was analyzed by cell treatment with test compounds for 48 h using cisplatin as a positive control. The cells (105 cells) were collected by trypsinization and washed twice with ice-cold PBS (pH 7.4). The cells were resuspended in 2 mL of 60% ice-cold ethanol and incubated at 4 °C for 1 h for fixation. The fixed cells were washed twice with PBS and re-suspended in 1 mL of PBS containing 50 µg/mL RNAase A and 10 µg/mL propidium iodide (PI). After 20 min of incubation in the dark at 37 C, the cells were analyzed for DNA contents using flow cytometry analysis using an FL2 (λex/em 535/617 nm) signal detector (ACEA Novocyte™ flowcytometer, ACEA Biosciences Inc., San Diego, CA, USA). For each sample, 12,000 events were acquired. The cell cycle distribution was calculated using ACEA NovoExpress™ software (ACEA Biosciences Inc., San Diego, CA, USA) [16].

### 2.6. In Silico Studies

#### 2.6.1. Ligand File Preparation

Propranolol, a beta-blocker drug, was downloaded as sdf to be in its minimized energy form from the Pubchem database (https://pubchem.ncbi.nlm.nih.gov/ (accessed on 20 January 2023)). This step was carried out to prepare the drug file in the required form to be used in the docking studies with different enzymes.

#### 2.6.2. Preparation of Enzyme Files

Different melanocyte receptors involved in melanoma were investigated and then downloaded from either the Swiss model (https://swissmodel.expasy.org/ (accessed on 21 January 2023)) [17,18] or the protein database bank (https://www.rcsb.org/ (accessed on 20 January 2023)) in the form of pdb files. Then, the files were refined by removing the corresponding co-crystal ligand from each enzyme file to obtain the enzyme-only files in pdb format using PyMol software version 2.2.3.

#### 2.6.3. Molecular Docking

The molecular docking of propranolol into different melanocyte receptors involved in melanoma was carried out using a CB-Dock docking server (http://clab.labshare.cn/cb-dock/php/index.php (accessed on 2 March 2023)) [19,20]. Drug-receptor interaction files for each receptor were downloaded separately in mol2 format as recommended by the used server. The studied receptors were: (a) G-protein coupled receptors, e.g., corticotrophin-releasing hormone receptor (CRH-R1), frizzled receptors, melanocortin receptors, melatonin receptors, metabotropic glutamate; (b) growth factors, e.g., ckit; (c) endothelin receptors; (d) death receptors (Fas death); (e) vascular endothelial growth factors receptors, e.g., VEGFR-1; and (f) vitamin D3 receptors.

#### 2.6.4. Interaction of Propranolol and Different Melanocyte Receptors

The interaction between each receptor individually and propranolol was visualized using Discovery Studio-19 software version 19.1.0.18287. The studied interactions included conventional H-bonding, aromatic ring center, hydrophobic interaction, covalent bond, perpendicular and parallel pi-stacking, charge center, etc. The receptor–propranolol binding interactions were assessed through calculations of binding free energy (DG).

### 2.7. Statistics

All experiments were conducted in triplicates and all data were reported as the mean ± SD. The GraphPad Prism 6 software (San Diego, CA, USA) was used to analyze the statistically significant difference using one-way analysis of variance (ANOVA) and Tukey post-test (* *p* < 0.05, ** *p* < 0.01, *** *p* < 0.001 and **** *p* < 0.0001).

## 3. Results and Discussion

### 3.1. Preparation of Propranolol-Loaded Trehalosome

#### 3.1.1. Analysis of Responses

**Figure 1:**
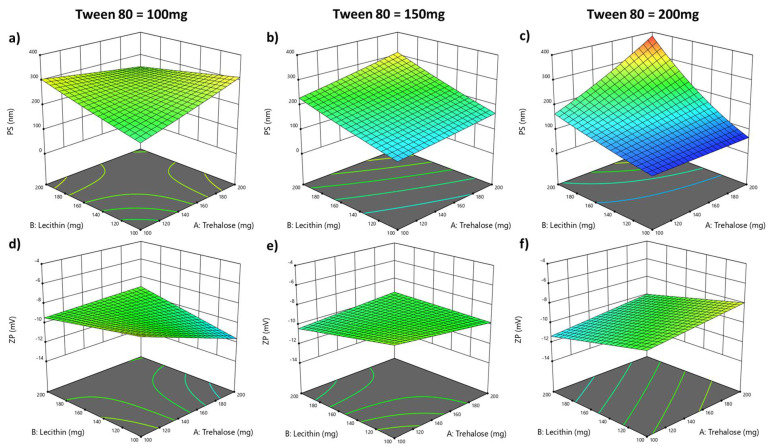

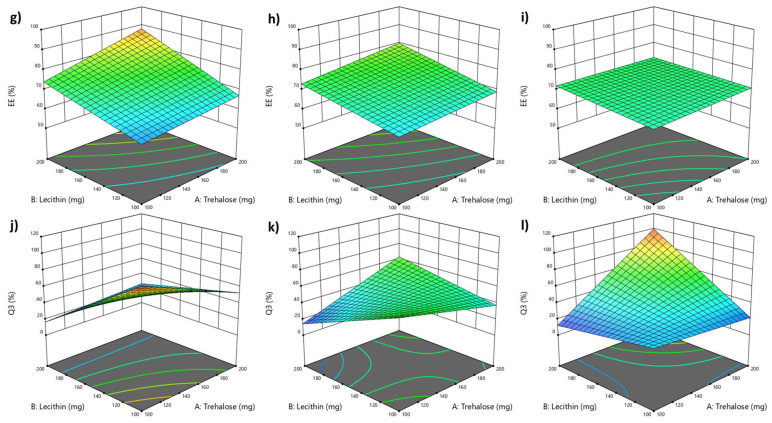
3D plots of different responses for the prepared trehalosome formulations: (**a**–**c**) particle size, (**d**–**f**) surface charge, (**g**–**i**) entrapment efficiency, and (**j**–**l**) dissolution percentage after 3 h. Color code: The color scale started from blue as a low value and increased gradually through green till reached red color for high value.

Different formulations were prepared according to the composition demonstrated in Table 1. In total, 23 factorial designs were created by Design Expert software. The experimental runs were 24 in order to consider the effect of three factors at different levels. As presented in Table 3, the formula was significantly affected by the levels of different factors and the interaction between them. The design used shows a promising fit to the design space, as all adequate precision values exceed four. Additionally, the adjusted R^2^ and predicted R^2^ are similar, which displays the consistency of the model. The impact of the factors on the responses was analyzed, and the findings are presented in 3D diagrams as shown in Figure 1.

#### 3.1.2. Particle Size

The prepared PTH formulations were evaluated via different techniques in which particle size was considered as one of the critical factors for the optimization of PTHs. Smaller particle size usually provides a good surface area to encapsulate drugs such as propranolol [21]. The data show that the obtained PTH sizes ranged from 65 up to 386.3 nm (Figure 1a–c). Three factors have a significant effect on particle size. Increasing trehalose and lecithin led to an increase in particle size, while increasing Tween 80 led to a decrease in the particle size, as shown in Table 4, where the *p* values were <0.0001. Moreover, the interaction of the second and third factors caused a significant increase in particle size (*p* values < 0.0001), as shown in Figure 1a–c. It was reported by Ochi et al. that lecithin hydrophobicity enlarges the vesicle dimension [22]. The incorporation of Tween 80 in PTHs causes a decrease in particle size due to steric repulsion rendered by surfactant molecules, which prevents or minimizes the aggregation of the vesicles [23].

#### 3.1.3. Surface Charge

The zeta potential is another parameter that affects the stability of nanocarriers, where high negativity or positivity of the surface charge can form a stable colloidal system [21,24]. It was found that the surface charge was not influenced by the three factors alone, but the interaction between trehalose and lecithin beside trehalose and Tween 80 were significant where *p*-values were 0.0121 and 0.0242, respectively, as shown in Table 4. The surface charges of the PTH formulation values ranged from −5.9 mV up to −12.8 mV, as shown in Figure 1d–f. Their surface’s negative charge indicates that most of the samples are physically stable where the negative charge will prevent aggregation. Furthermore, the presence of Tween 80 could prevent the agglomeration of nanoparticles through the steric hindrance effect [25]. The combination of small particle size and low negative surface charge enabled high dermal penetration by trehalosomes, which is appropriate for treating skin cancer [26].

#### 3.1.4. Entrapment Efficiency

Entrapment efficiency is usually measured to determine the encapsulation ability of the nanocarrier. The EE percentage ranged from 57% up to 92.5%, as shown in Figure 1g–i. It has been found that increasing trehalose and lecithin have a significant influence on the EE% results, as shown in Table 4, with *p*-values of 0.0013 and <0.0001, respectively. Furthermore, the interaction between trehalose and the two other factors negatively affects the EE%. According to Table 4, increasing the Tween 80 level showed an insignificant effect on EE%. Therefore, the formulations with a low level of Tween 80 showed high EE%.

#### 3.1.5. Dissolution after 3 h

The release profile of PPL was also conducted to study the ability of the nanocarrier system to control drug release. The three factors significantly affected the Q3, as shown in Figure 1j–l and Table 4. The release after 3 h ranged from 12% to 100%, depending on the level of the three factors. As displayed in Table 4, trehalose has a significant (*p* < 0.0001) positive effect on the drug released. This could be attributed to the high solubility of trehalose, which could provide channels in the bilayer structure of trehalosomes, allowing the drug release. On the other hand, lecithin and Tween 80 had a significant negative effect (*p* < 0.0001) on the drug released.

#### 3.1.6. Optimization

**Figure 2:**
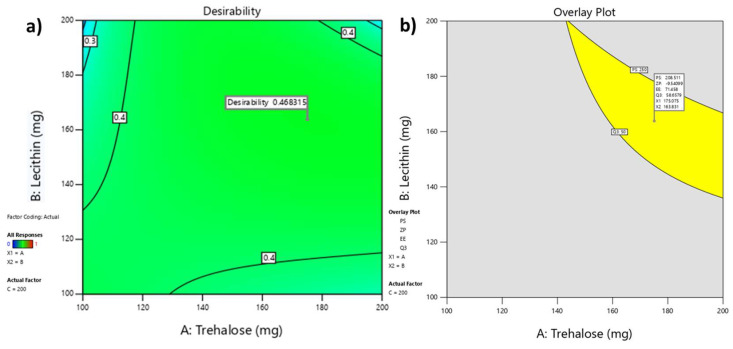
(**a**) Desirability plot obtained and (**b**) design space.

Numerical and graphical analyses were conducted to determine the best formula for achieving the minimum size and maximum surface charge, EE%, and Q3 (Table 3). The results of both optimizations were similar, indicating the reliability of the chosen model (Figure 2). The best optimum formula has a desirability of 0.468. This optimum formula consisted of trehalose (175 mg), lecithin (164 mg), and Tween 80 (200 mg), as shown in Table 5. According to program analysis, this formula should have a size of 245 nm, a charge of −9 mV, an EE% of 68%, and a Q3 of 62%. This formula was then prepared and characterized to confirm the accuracy of the predictions through experimental methods as shown in Table 5.

#### 3.1.7. Characterization of Optimum Formula

**Figure 3:**
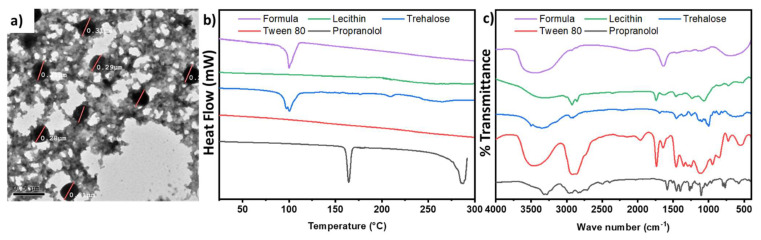
(**a**) TEM micrograph of the optimized trehalosome formulation using Joel JEM 1230, Tokyo, Japan, (**b**) DSC thermogram of the optimum formula and each component using DSC50, Shimadzu, Japan, and (**c**) FTIR spectrum of the optimum formula and each component using FTIR −8400, Shimadzu, Japan.

A TEM micrograph of the optimum formula showed spherical non-aggregated vesicles with particle sizes ranging from 280 to 310 nm, as shown in Figure 3a. In addition, the results indicate that the particle size of the developed vesicles is consistent with the size predicted by the design. To confirm the drug encapsulation, DSC thermograms were measured, as shown in Figure 3b. The propranolol thermogram showed an endothermic peak at 163.8 °C, corresponding to its melting point. This finding was in agreement with the previously reported melting point of propranolol [27]. The melting point of trehalose was around 100, which was in agreement with the previously reported melting point [28]. The melting point of the prepared formula showed the disappearance of the drug melting point, which confirms the drug diffusion in the prepared vesicles [29,30]. Furthermore, Figure 3c demonstrated the FTIR spectrum of the prepared formula. The drug showed a characteristic absorption peak at 2965 cm^−1^ due to the presence of a secondary amine group and 3283 cm^−1^ due to the hydroxyl group (secondary); the aryl alkyl ether displayed a stretching band at 1267.27 cm^−1^ and a peak at 798 cm^−1^ due to a-substituted naphthalene [31]. The spectrum of the prepared formula showed the characteristic absorption peaks of the drug and vesicle components, which emphasize the success of the trehalosome assembly and drug encapsulation.

### 3.2. In Vitro Cell Study

#### 3.2.1. Cytotoxicity Cell Study

**Figure 4:**
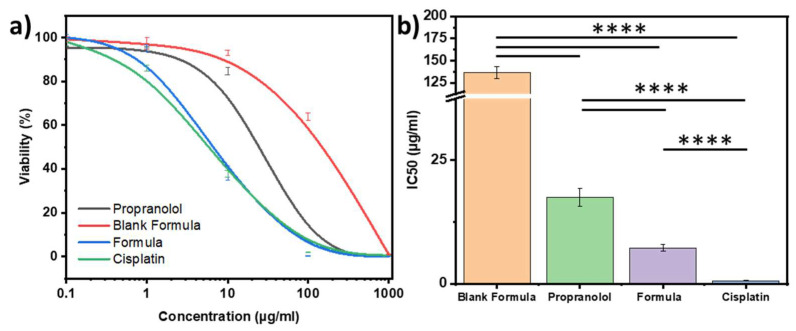
Cytotoxicity cell study: (**a**) cell viability percentage was determined using BMG LABTECH^®^-FLUOstar Omega microplate reader and (**b**) IC_50_ of blank formula, free propranolol, loaded formula and cisplatin (n = 3, **** *p* < 0.0001).

The study evaluated the cytotoxicity of free propranolol, blank and loaded formula, and cisplatin against an A 375 human melanoma cell line using the SRB assay. After 72 h of treatment with different concentrations (0.1–1000 μg/mL) of the four test groups, the cell viability was measured as shown in Figure 4a. The results showed that the cell viability of the A 375 human melanoma cell line decreased in a dose-dependent manner in response to treatment by either free propranolol, blank and loaded formula, or cisplatin. Treatment with cisplatin resulted in a greater decrease in the cell viability percentage, with IC50 0.59 μg/mL compared to treatment with other groups, which could be attributed to the potency of cisplatin as a standard drug. However, cisplatin still has serious side effects, which motivates researchers to find alternative effective drugs with lower side effects [32]. On the other hand, propranolol as a beta-blocker drug was previously reported as a promising anticancer drug. Beta-blockers, particularly propranolol, have been found to significantly improve the outlook of melanoma patients. Propranolol works by reducing the growth of blood vessels in the tumor, as well as the proliferation, invasiveness, and immune suppression of tumor cells. Studies have shown that only β3-adrenoceptors, rather than β2-adrenoceptors, were increased in peripheral blood mononuclear cells under hypoxia. These receptors were selectively found in immune cell sub-populations, including Treg, MDSC, and NK cells, which increased the number and cytotoxicity of NK and CD8 cells. Catecholamines may slow down the progression of melanoma, and β-blockers may have untapped potential as a treatment for melanoma, both as a preventive measure and as an adjuvant to other targeted and immune therapies [1,33]. In the current study, the IC50 of free propranolol was 17.48 μg/mL, as shown in Figure 4b. The encapsulation of propranolol showed a significant decrease (*p* < 0.0001) in IC50 to reach 7.26 μg/mL. This confirmed that the required dose of propranolol could be decreased almost to half when being incorporated within trehalosomes instead of being administered as a free drug. The blank formula achieved IC50 136.7 μg/mL, which confirmed that the synergistic effects of both propranolol and trehalosmoes led to a decrease in the IC50. To understand the cytotoxicity mechanism of free and encapsulated propranolol, further investigations were conducted, as shown in the next section.

#### 3.2.2. Annexin-V-FITC Apoptosis Assay

**Figure 5:**
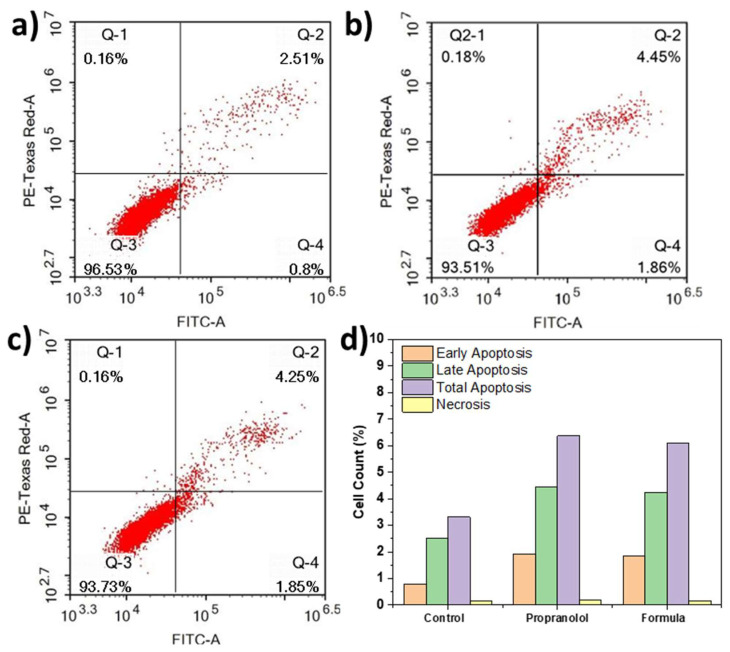
Apoptotic/necrotic assay by flow cytometry (ACEA Biosciences Inc., San Diego, CA, USA) for human melanoma cell line incubated with (**a**) untreated cells, (**b**) free propranolol, (**c**) propranolol-loaded trehalosomes, and (**d**) percentage of cells death of early apoptosis, late apoptosis, and necrosis.

To determine how different treatments inhibit the proliferation of human melanoma cell lines, an Annexin V/propidium iodide assay kit was utilized. The assay measured early apoptosis (Q4), late apoptosis (Q2), necrosis (Q1), and cell viability (Q3) after a 48 h incubation period, as shown in Figure 5a–c. The experiment included untreated human melanoma cell lines as a control, as well as cells treated with free and encapsulated propranolol. In Figure 5d, the two treatments were found to significantly increase early and late apoptosis compared to the control, which is consistent with previous reports of the antiproliferative activity of propranolol [34]. The cytotoxicity of free propranolol was mainly due to the additive effect of early and late apoptosis, while necrosis played a minor role. Similarly, propranolol-loaded trehalosomes showed cytotoxicity due to the additive effect of early and late apoptosis, with a lower contribution from necrosis. Interestingly, apoptosis was similar in the case of free and encapsulated propranolol, indicating the trehalosomes’ ability to induce programmed death in cancer cells [15]. To learn more about the nature of the cytotoxicity mechanism of the prepared formula, a cell cycle analysis was conducted in the next section.

#### 3.2.3. Cell Cycle Analysis

**Figure 6:**
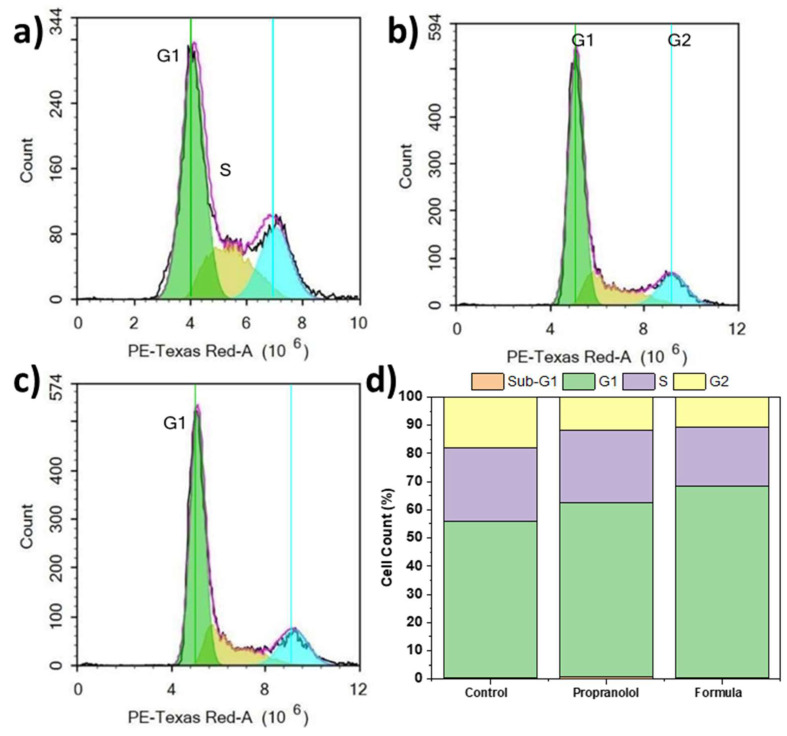
Cell cycle analyzed by flow cytometry (ACEA Biosciences Inc., San Diego, CA, USA) for human melanoma cell line incubated with (**a**) untreated cells, (**b**) free propranolol, (**c**) propranolol-loaded trehalosomes (Color code: Green represent G1 phase population, Yellow represent S phase population, and Blue represent G2 phase population), and (**d**) percentage of cells cycle analysis.

This experiment investigated different phases of the cell cycle, including G0-G1 (apoptosis or non-dividing cells), S (DNA synthesis phase), and G2-M (proliferation or mitosis). An untreated human melanoma cell line was used as a control, and cell cycle analysis of free and encapsulated propranolol was conducted using nuclear propidium iodide staining and flow cytometry as shown in Figure 6a–c. The results show that free propranolol mainly suppressed the human melanoma cell line in the G1 phase, with 61.8% of cells accumulating in this phase. Propranolol also demonstrated the ability to suppress cancer cells in the S and G2 phases, with 25.7% and 15.2% accumulation, respectively. This is consistent with previous reports of propranolol’s ability to de-regulate cell cycle progression and induce caspase three- and nine-mediated apoptosis [35]. Similarly, propranolol-loaded trehalosomes suppressed the human melanoma cell line in the G1 phase, followed by the S phase and then the G2 phase, with 67.8%, 21.1%, and 15.7% accumulation, respectively. This can be attributed to the presence of trehalose within the nanocarrier structure [10]. There was a marked buildup in the cells in the G1 phase by nearly 1.2 times for free propranolol and propranolol-loaded trehalosome-treated cells when compared to the control, as shown in Figure 6d. Moreover, a low concentration of cells transitioned to the S and G2 phases. Moreover, there is a noticeable increase in the sub G1 phase in cells treated with propranolol and propranolol-loaded trehalosomes by two folds due to apoptosis induction, as displayed in Figure 6d. These results demonstrated the G1 phase cell cycle arrest for the propranolol and propranolol-loaded trehalosome. Furthermore, the current results confirmed the role of trehalosomes in the prevention of malignant melanoma cell proliferation, which is attributed to both propranolol and propranolol-loaded trehalosomes [10].

### 3.3. In Silico Studies

#### 3.3.1. Molecular Docking between Propranolol and Different Melanocytes Receptors

A CB-Dock docking server (http://clab.labshare.cn/cb-dock/php/index.php (accessed on 2 March 2023)) [19,20] was used to calculate Gibbs free energy. This was carried out to study the individual strengths of the interactions between propranolol on the one hand and different studied melanocyte receptors on the other hand. Different melanocyte receptors that have been reported in the literature to be involved in melanoma were selected [36]. It was observed that propranolol has a high binding affinity to all of the studied enzymes, as the Gibbs free energy ranged from—6.8 in the case of endothelin and the frizzled receptors up to—8.6 in the case of the vitamin D3 receptors, as shown in Table 6. This was found to be in accordance with what has been reported previously in the literature that states that beta-blockers possess anti-cancer activity in melanoma patients [33,37,38].

#### 3.3.2. Visualization of Interaction between Propranolol and Different Melanocyte Receptors

The individual interactions between propranolol and different melanocyte receptors were visualized using Discovery Studio-19 software to determine their implications in melanomas.

##### G-Protein Coupled Receptors

Interaction visualizations are demonstrated in the following sections.

- *Corticotrophin-Releasing Hormone Receptor (CRH-R1)*

**Figure 7:**
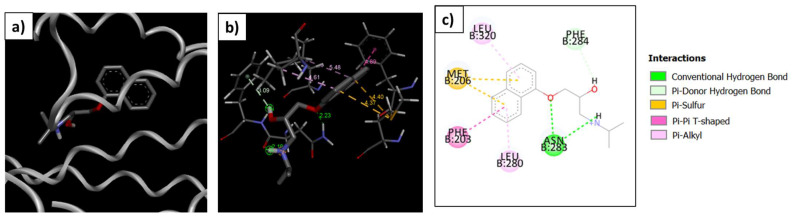
(**a**) Overview of insertion of propranolol into corticotrophin-releasing hormone receptor (CRH-R1), (**b**) 3D modeling of propranolol interaction with corticotrophin-releasing hormone receptor (CRH-R1), and (**c**) 2D diagrams of the interactions of propranolol and corticotrophin-releasing hormone receptor (CRH-R1).

Both melanocytes and melanoma cells have been reported to express CRH-R1 responding to CRH peptides via activating cAMP, IP3, and Ca-mediated pathways in order to modify the phenotype of melanocytes. CRH-R1 regulates cell proliferation and differentiation. As shown in Figure 7 and Table 7, propranolol could interact with CRH-R1 via eight interactions: one pi-pi stacking with PHF (B: 203), two hydrophobic (alkyl–sulfur) interactions with MET (B:206), two hydrophobic (alkyl–alkyl) interactions with LEU (B: 280) and LEU (B: 320), and three hydrogen bonds, two with ASN (B: 283) and one with PHE (B: 284). These interactions suggest that propranolol can be a good candidate as a CRH-R1 antagonist, and consequently as a cell proliferation inhibitor in malignant tumors, including melanoma. This is found to be in good agreement with what has been reported in the literature concerning CRH-R1 antagonists as anti-cancer agents, including melanoma [36,39].

- *Frizzled receptor*

**Figure 8:**
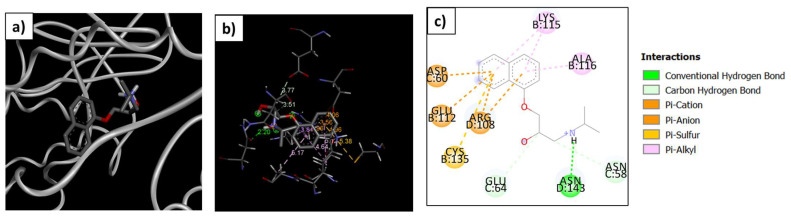
(**a**) Overview of insertion of propranolol into frizzled receptor, (**b**) 3D modeling of propranolol interaction with frizzled receptor, and (**c**) 2D diagrams of the interactions of propranolol and frizzled receptor.

Frizzled receptors initiate signals resulting in β-catenin stabilization upon Wnt binding. This leads to regulating the genes responsible for tumorigenesis, e.g., cyclin D1, c-Myc, and matrix metalloproteinase. Hence, blocking frizzled receptors would enhance malignancy inhibition. As shown in Figure 8 and Table 8, propranolol could interact with frizzled receptors through ten interactions: two hydrophobic (alkyl–anion) interactions with GLU (B: 112) and ASP (C: 60), two hydrophobic (alkyl–alkyl) interactions with LYS (B: 115) and ALA (B: 116), one hydrophobic (alkyl–sulfur) with CYS (B: 135), three hydrogen bonds with ASN (C: 58), GLU (C: 64), and ASN (D: 143), and two hydrophobic (alkyl–cation) interactions with ARG (D: 108). These interactions suggest that propranolol can be a good candidate as a frizzled antagonist and consequently as a melanoma suppressor. This is found to be in concurrence with what has been reported in the literature concerning frizzled antagonists as anti-melanoma agents [36,40,41].

- *Melanocortin receptor*

**Figure 9:**
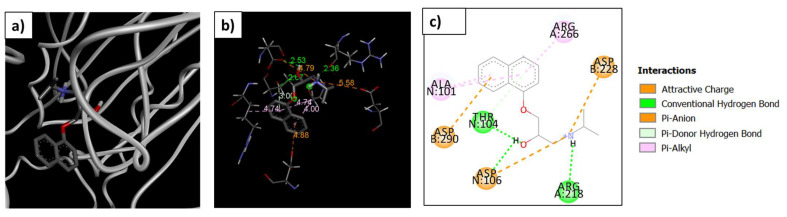
(**a**) Overview of insertion of propranolol into melanocortin receptor, (**b**) 3D modeling of propranolol interaction with melanocortin receptor, and (**c**) 2D diagrams of the interactions of propranolol and melanocortin receptor.

Melanocortin receptors are involved in gene polymorphism, which is responsible for pigmentation and sensitization toward UV radiation. Overexpression of these receptors would consequently lead to the propagation of melanoma. Hence, the blockage of these receptors would help in minimizing melanoma propagation. As shown in Figure 9 and Table 9, propranolol could interact with melanocortin receptors through eight interactions: two hydrogen bonds with ARG (A: 218) and THR (N: 104); three hydrophobic (alkyl–alkyl) interactions, one with ARG (A: 266) and two with ALA (N: 101); and three hydrophobic (alkyl–anion) with ASP (B: 228), ASP (B: 290), and ASP (N: 106). These interactions suggest that propranolol can be a good candidate as a melanocortin antagonist and consequently as a melanoma suppressor. This is found to be in alignment with what has been reported in the literature concerning melanocortin antagonists as anti-melanoma agents [36,42].

- *Melatonin receptor*

**Figure 10:**
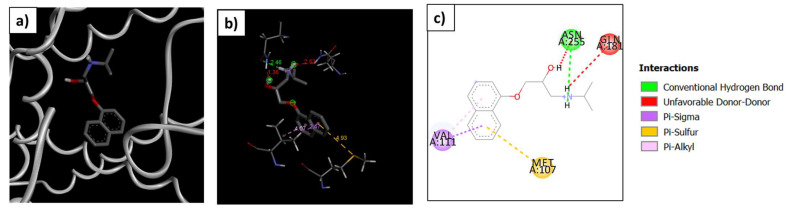
(**a**) Overview of insertion of propranolol into melatonin receptor, (**b**) 3D modeling of propranolol interaction with melatonin receptor, and (**c**) 2D diagrams of the interactions of propranolol and melatonin receptor.

Melatonin agonists have been reported for their efficiency in producing melatonin as a protectant against external stress factors that may lead to melanoma. Hence, the interaction between propranolol and melatonin receptors could lead to participation in suppressing melanoma. As shown in Figure 10 and Table 10, propranolol could interact with melanocortin receptors through six interactions: one hydrophobic (alkyl–sulfur) interaction with MET (A: 107), a pi-sigma bonding and hydrophobic (alkyl–alkyl) interaction with VAL (A: 111), a donor–donor interaction with GLN (A: 181), and a hydrogen bonding and a donor–donor interaction ASN (A: 255). This suggests that propranolol can be a good activator for melatonin receptors. This is found to be similar to what has been reported in the literature concerning the role of melatonin receptors in melanoma treatment [43,44].

- *Metabotropic glutamate receptor*

**Figure 11:**
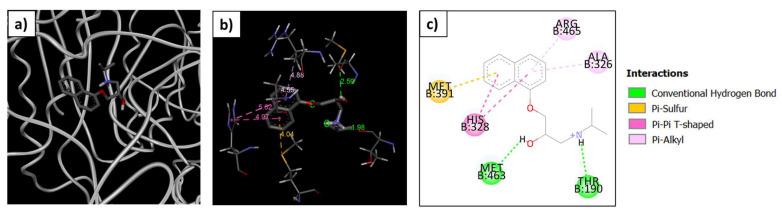
(**a**) Overview of insertion of propranolol into metabotropic glutamate receptor, (**b**) 3D modeling of propranolol interaction with metabotropic glutamate receptor, and (**c**) 2D diagrams of the interactions of propranolol and metabotropic glutamate receptor.

Metabotropic glutamate receptor 1 (GRM1) is overexpressed in the incidence of melanoma. Hence, its inhibition suppresses melanoma progression. As shown in Figure 11 and Table 11, propranolol could interact with metabotropic glutamate receptors through seven interactions: two hydrogen bonds with THR (B: 190) and MET (B: 463), two hydrophobic (alkyl–alkyl) interactions with ALA (B: 326) and ARG (B: 465), two pi-pi stacking interactions with HIS (B: 328), and one hydrophobic (alkyl–sulfur) interaction with MET (B: 391). This is found to be in conformity with what has been reported in the literature concerning metabotropic glutamate antagonists as anti-melanoma agents [36].

##### Growth Factors

**Figure 12:**
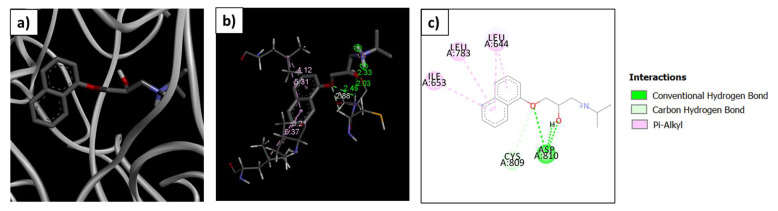
(**a**) Overview of insertion of propranolol into growth factors, (**b**) 3D modeling of propranolol interaction with growth factors, and (**c**) 2D diagrams of the interactions of propranolol and growth factors.

C-Kit (CD117) is a growth factor for melanocytes that affects their proliferation, melanogenesis, and survival. Thus, the overexpression of c-kit in melanoma should be targeted with antagonists to suppress that propagation. As shown in Figure 12 and Table 12, propranolol could interact with C-Kit via eight interactions: four hydrophobic (alkyl–alkyl) interactions, two with LEU (A: 644), one with ILE (A: 653), and one with LEU (A: 783); and four hydrogen bonds, one with CYS (A: 809) and three with ASP (A: 810). This is found to be in good agreement with what has been reported in the literature concerning C-Kit antagonists as anti-melanoma agents [36,45].

##### Endothelin (EDN) Receptors

**Figure 13:**
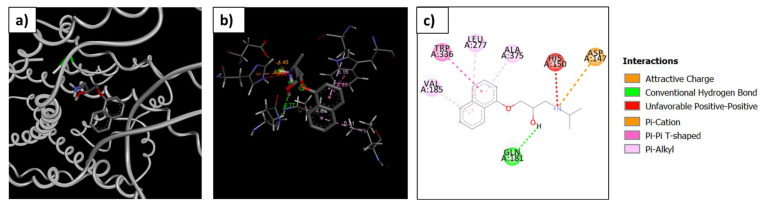
(**a**) Overview of insertion of propranolol into endothelin (EDN) receptors, (**b**) 3D modeling of propranolol interaction with endothelin (EDN) receptors, and (**c**) 2D diagrams of the interactions of propranolol and endothelin (EDN) receptors.

In melanoma, EDN has been found to stimulate the down-regulation of E-cadherin expression and the up-regulation of N-cadherin, leading to an increase in tumor proteolytic activity, which in turn provokes melanoma cell adhesion, proliferation, and migration. Hence, blocking EDN results in decreasing melanoma cell viability as well as DNA synthesis while increasing melanoma apoptosis. As shown in Figure 13 and Table 13, propranolol could interact with EDN via seven interactions: one hydrophobic (alkyl–cation) interaction with ASP (A: 147), one positive–positive interaction with HIS (A: 150), a hydrogen bond with GLN (A: 181), three hydrophobic (alkyl–alkyl) interactions with VAL (A: 185), LEU (A: 277), and ALA (A: 375), and pi-pi stacking with TRP (A: 336). This is found to be in concurrence with what has been reported in the literature concerning EDN antagonists as anti-melanoma agents [36,46].

##### Death Receptors

**Figure 14:**
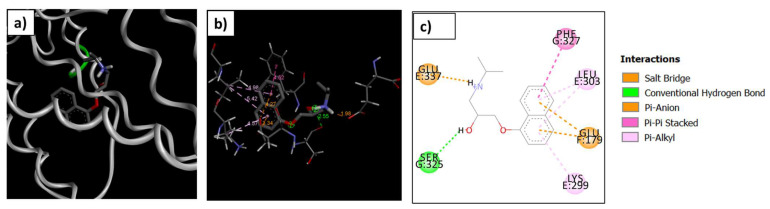
(**a**) Overview of insertion of propranolol into death receptors, (**b**) 3D modeling of propranolol interaction with death receptors, and (**c**) 2D diagrams of the interactions of propranolol and death receptors.

Death receptors such as Fas (CD95) can be stimulated to trigger the cellular death of melanocytes, including melanoma. Hence, agents that are capable of triggering these death receptors have proven their efficiency in the treatment of metastatic melanoma. As shown in Figure 14 and Table 14, propranolol could interact with Fas receptors via eight interactions: three hydrophobic (alkyl–alkyl) interactions, one with LYS (E: 299) and two with LEU (E: 303); one hydrophobic (alkyl–cation) interaction with GLU (E: 337); two hydrophobic (alkyl–anion) interactions with GLU (F: 179); one hydrogen bond with SER (G: 325); and one pi–pi stacking with PHF (G: 327). This is found to be in alignment with what has been reported in the literature concerning death receptor activators as anti-melanoma agents [36,47].

##### Vascular Endothelial Growth Factors

**Figure 15:**
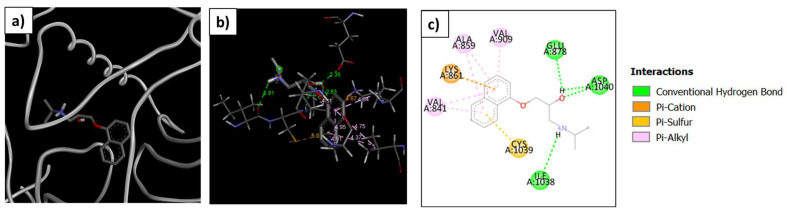
(**a**) Overview of insertion of propranolol into vascular endothelial growth factors, (**b**) 3D modeling of propranolol interaction with vascular endothelial growth factors, and (**c**) 2D diagrams of the interactions of propranolol and vascular endothelial growth factors.

Vascular endothelial growth factors such as the VEGFR-1 receptor possess a tyrosine kinase activity capable of activating signaling pathways that could alter patterns of gene expression in order to enhance melanocyte proliferation. Hence, the blockage of these factors is considered a promising target in suppressing melanoma. As shown in Figure 15 and Table 15, propranolol could interact with VEGFR-1 via eleven interactions: five hydrophobic (alkyl–alkyl) interactions, two with VAL (A: 841), two with ALA (A: 859), and one with VAL (A: 909); one hydrophobic (alkyl–cation) with LYS (A: 861); four hydrogen bonds, one with GLU (A: 878), one with ILE (A: 1038), and two with ASP (A: 1040); and one hydrophobic (alkyl–sulfur) interaction with CYS (A: 1039). This is found to be in conformity with what has been reported in the literature concerning VEGFR receptor inhibitors as anti-melanoma agents owing to their antiproliferative and anti-angiogenic activities [35,47,48].

##### Vitamin D3 Receptors

**Figure 16:**
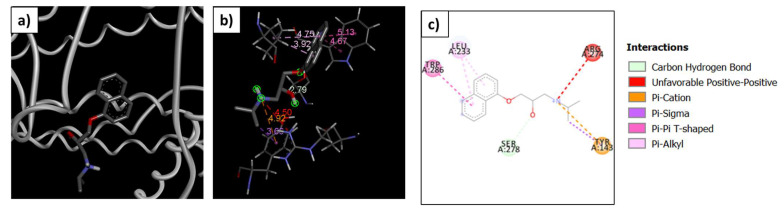
(**a**) Overview of insertion of propranolol into vitamin D3 receptors, (**b**) 3D modeling of propranolol interaction with vitamin D3 receptors, and (**c**) 2D diagrams of the interactions of propranolol and vitamin D3 receptors.

Besides their involvement in calcium metabolism, vitamin D3 receptors play a role in melanocyte regulation and apoptosis. Hence, it has been reported that vitamin D derivatives or vitamin D3 receptor triggers could aid in melanoma treatment. As shown in Figure 16 and Table 16, propranolol could interact with vitamin D3 receptors via eight interactions: one hydrophobic (alkyl–cation) interaction and one pi–sigma interaction with TYR (A: 143), two hydrophobic (alkyl–alkyl) interactions with LEU (A: 233), one positive–positive interaction with ARG (A: 274), one hydrogen bond with SER (A: 278), and two pi-pi stacking interactions with TRP (A: 286). This is found to be in alignment with what has been reported in the literature concerning vitamin D3 receptor-triggering agents as anti-melanoma agents [35,49,50].

It is inferred from the in silico studies that the highest influential receptors in melanoma are vitamin D receptors, CRH-R1, VEGFR 1, and c-Kit, in descending order. This has been detected by assessing the binding energy that showed −8.6, −8.5, −8.4, and −8.3, respectively. This was also confirmed through detecting high bond interactions through discovery studio visualization. Interestingly, the results of in silico studies were found to match and support the results of experimental apoptotic and cell cycle analysis reported in Section 3.2.2 and Section 3.2.3, respectively.

For instance, the vitamin D receptor, which is responsible for melanocyte regulation and apoptosis, could be triggered using propranolol, leading to melanoma apoptosis. This is found to be in good agreement with apoptotic results through caspase enzyme inhibition. In addition, CRH-R1, which was reported for its proliferative activity, has also been found to be strongly inhibited by propranolol. This is found to be in good accordance with what has been reported in cell cycle analysis. Furthermore, in silico studies showed that both c-Kit and VEGFR 1, which are responsible for mutagenesis, can be blocked using propranolol, resulting in anti-proliferation activity. This also supports the experimental results above. Finally, experimental and in silico studies collectively suggest that propranolol can be used as a promising antiproliferative and apoptotic agent in melanoma treatment.

## 4. Conclusions

Propranolol-loaded trehalosomes were developed using the ethanol injection method. A factorial design was used to select the optimum formula, where trehalose, lecithin, and Tween 80 levels were studied to achieve minimum size and maximum charge, EE%, and Q3. The optimized formula was investigated using TEM, DSC, and FTIR. Cell studies were carried out against the human melanoma cell line to measure cytotoxicity, apoptosis/necrosis, and cell cycle arrest. The results show that the selected formula has good cytotoxicity compared to the free drug due to the synergistic effect of the drug and the designed carrier, which make it a good candidate as an antiproliferative agent for treating skin cancer. Therefore, the future plan is to study the efficacy of the combination of the selected formula with the traditional chemotherapeutic agent using an in vivo animal model to decrease the chemotherapeutic agent dose, which in turn will decrease the side effects.

## Figures and Tables

**Table 1:** Composition of the prepared PPL-loaded trehalosomes.

Code	Drug (mg)	Trehalose (mg)	Lecithin (mg)	Tween 80 (mg)
**F1**	100	100	200	200
**F2**	100	200	100	200
**F3**	100	200	200	100
**F4**	100	100	100	200
**F5**	100	100	200	100
**F6**	100	200	100	100
**F7**	100	100	100	100
**F8**	100	200	200	200

**Table 2:** Factors of 2^3^ factorial design for PPL-loaded trehalosome formulations with model statistics and optimization.

Factors	Levels		Optimized Formula
	Low	High	
**X1: Trehalose (mg)**	100	200	175
**X2: Lecithin (mg)**	100	200	164
**X3: Tween 80 (mg)**	100	200	200

**Table 3:** Responses of 2^3^ factorial design for PPL-loaded trehalosome formulations with model statistics.

Responses	Desirability Constraints	R^2^	Adjusted R^2^	Predicted R^2^	Adequate Precision	Significant Factors
**Y1: Size (nm)**	Minimize	0.999	0.999	0.998	126.99	X_1_, X_2_, X_3_ X_1_X_2_, X_1_X_3_, X_2_X_3_
**Y2: Charge (mV)**	Maximize	0.604	0.430	0.109	5.00	X_1_X_3_, X_2_X_3_
**Y3: EE (%)**	Maximize	0.871	0.814	0.709	13.58	X_1_, X_2_ X_1_X_3_, X_2_X_3_
**Y4: Dissolution 3 h (%)**	Maximize	0.993	0.990	0.984	44.43	X_1_, X_2_, X_3_ X_1_X_2_, X_1_X_3_, X_2_X_3_

**Table 4:** A coefficient table for the responses of the different PTH formulations.

	Intercept	X1	X2	X3	X1 × X2	X1 × X3	X2 × X3	X1 × X2 × X3
**PS**	14.286	**1.0563**	**2.15245**	**−1.95776**	**0.203671**	**0.545638**	**1.7552**	**1.55436**
***p*** **-values**		**<0.0001**	**<0.0001**	**<0.0001**	**<0.0001**	**<0.0001**	**<0.0001**	**<0.0001**
**ZP**	−9.52713	−0.092875	−0.412875	−0.052125	0.537875	**0.747125**	**−0.657875**	−0.492125
***p*** -values *		0.7298	0.1378	0.8461	0.0587	**0.0121**	**0.0242**	0.0810
**EE**	71.875	**2.625**	**4.625**	−0.875	0.875	**−2.125**	**−3.625**	−1.375
***p*** -values *		**0.0013**	**<0.0001**	0.2146	0.2146	**0.0063**	**<0.0001**	0.0593
**Q3**	45.115	**5.18**	**−6.375**	**−3.6275**	**19.245**	**14.2925**	**20.9025**	**5.1675**
***p*** -values *		**<0.0001**	**<0.0001**	**<0.0001**	**<0.0001**	**<0.0001**	**<0.0001**	**<0.0001**

* *p*-value shading: *p* < 0.05; 0.05 ≤ *p* < 0.1; *p* ≥ 0.1.

**Table 5:** Optimization of 2^3^ factorial design for PPL-loaded trehalosome formulations.

Responses	Observed Values	Predicted Values	Bias %
**Y1: Size (nm)**	245	208.6	14.80%
**Y2: Charge (mV)**	−9	−9.5	5.50%
**Y3: EE (%)**	68	71.4	5%
**Y4: Dissolution 3 h (%)**	62	58.6	5.40%

**Table 6:** Gibbs free energy shows the interaction between propranolol and different melanocyte receptors.

Propranolol	
Enzyme	Gibbs Free Energy
Endothelin	−6.8
Frizzled	−6.8
Fas Death	−7
Melanocortin	−7
Metabotropic Glutamate	−7.1
Melatonin	−7.9
Ckit	−8.3
VEGFR 1	−8.4
CRH-R1	−8.5
Vitamin D Receptor	−8.6

**Table 7:** A summary of types of interactions detected between propranolol and corticotrophin-releasing hormone receptor (CRH-R1).

Amino Acid	Bond or Interaction	Distance (A°)
PHF (B: 203)	Pi-Pi Stacking	4.89
MET (B: 206)	Hydrophobic (alkyl–sulfur)	4.37, 4.40
LEU (B: 280)	Hydrophobic (alkyl–alkyl)	5.48
ASN (B: 283)	Hydrogen Bonding	2.19, 2.23
PHE (B: 284)	Hydrogen Bonding	3.09
LEU (B:320)	Hydrophobic (alkyl–alkyl)	4.61

**Table 8:** A summary of types of interactions detected between propranolol and frizzled receptor.

Amino Acid	Bond or Interaction	Distance (A°)
GLU (B:112)	Hydrophobic (alkyl–anion)	4.96
LYS (B: 115)	Hydrophobic (alkyl–alkyl)	5.12
ALA (B:116)	Hydrophobic (alkyl–alkyl)	5.17
CYS (B:135)	Hydrophobic (alkyl–sulfur)	5.38
ASN (C: 58)	Hydrogen Bonding	3.51
ASP (C: 60)	Hydrophobic (alkyl–anion)	4.06
GLU (C: 64)	Hydrogen Bonding	3.77
ARG (D: 108)	Hydrophobic (alkyl–cation)	3.56, 3.96
ASN (D: 143)	Hydrogen Bonding	2.20

**Table 9:** A summary of types of interactions detected between propranolol and melanocortin receptor.

Amino Acid	Bond or Interaction	Distance (A°)
ARG (A: 218)	Hydrogen Bonding	2.36
ARG (A: 266)	Hydrophobic (alkyl–alkyl)	2.74
ASP (B: 228)	Hydrophobic (alkyl–anion)	2.58
ASP (B: 290)	Hydrophobic (alkyl–anion)	4.88
ALA (N: 101)	Hydrophobic (alkyl–alkyl)	4.00, 4.74
THR (N: 104)	Hydrogen Bonding	3.00
ASP (N: 106)	Hydrophobic (alkyl–anion)	4.79

**Table 10:** A summary of types of interactions detected between propranolol and melatonin receptor.

Amino Acid	Bond or Interaction	Distance (A°)
MET (A: 107)	Hydrophobic (alkyl–sulfur)	4.93
VAL (A: 111)	Pi-Sigma	2.47
	Hydrophobic (alkyl–alkyl)	4.07
GLN (A: 181)	Donor–Donor	2.53
ASN (A: 255)	Hydrogen Bonding	2.46
	Donor–Donor	1.36

**Table 11:** A summary of types of interactions detected between propranolol and metabotropic glutamate receptor.

Amino Acid	Bond or Interaction	Distance (A°)
THR (B: 190)	Hydrogen Bonding	1.98
ALA (B: 326)	Hydrophobic (alkyl–alkyl)	4.55
HIS (B: 328)	Pi-Pi Stacking	4.97, 5.62
MET (B: 391)	Hydrophobic (alkyl–sulfur)	4.04
MET (B: 463)	Hydrogen Bonding	2.59
ARG (B: 465)	Hydrophobic (alkyl–alkyl)	4.88

**Table 12:** A summary of types of interactions detected between propranolol and growth factors.

Amino Acid	Bond or Interaction	Distance (A°)
LEU (A: 644)	Hydrophobic (alkyl–alkyl)	4.12, 5.31
ILE (A: 653)	Hydrophobic (alkyl–alkyl)	5.21
LEU (A: 783)	Hydrophobic (alkyl–alkyl)	5.37
CYS (A: 809)	Hydrogen Bonding	2.88
ASP (A: 810)	Hydrogen Bonding	2.03, 2.33, 2.45

**Table 13:** A summary of types of interactions detected between propranolol and endothelin (EDN) receptors.

Amino Acid	Bond or Interaction	Distance (A°)
ASP (A: 147)	Hydrophobic (alkyl–cation)	4.27
HIS (A: 150)	Positive–Positive	4.53
GLN (A: 181)	Hydrogen Bonding	2.77
VAL (A: 185)	Hydrophobic (alkyl–alkyl)	4.83
LEU (A: 277)	Hydrophobic (alkyl–alkyl)	5.31
TRP (A: 336)	Pi-Pi Stacking	4.83
ALA (A: 375)	Hydrophobic (alkyl–alkyl)	5.15

**Table 14:** A summary of types of interactions detected between propranolol and death receptors receptors.

Amino Acid	Bond or Interaction	Distance (A°)
LYS (E: 299)	Hydrophobic (alkyl–alkyl)	4.57
LEU (E: 303)	Hydrophobic (alkyl–alkyl)	4.98, 5.42
GLU (E: 337)	Hydrophobic (alkyl–cation)	1.98
GLU (F: 179)	Hydrophobic (alkyl–anion)	3.34, 4.27
SER (G: 325)	Hydrogen Bonding	2.55
PHF (G: 327)	Pi-Pi Stacking	4.52

**Table 15:** A summary of types of interactions detected between propranolol and vascular endothelial growth factors receptors.

Amino Acid	Bond or Interaction	Distance (A°)
VAL (A: 841)	Hydrophobic (alkyl–alkyl)	4.37, 4.75
ALA (A: 859)	Hydrophobic (alkyl–alkyl)	4.91, 4.95
LYS (A: 861)	Hydrophobic (alkyl–cation)	4.67
GLU (A: 878)	Hydrogen Bonding	2.35
VAL (A: 909)	Hydrophobic (alkyl–alkyl)	4.11
ILE (A: 1038)	Hydrogen Bonding	2.91
CYS (A: 1039)	Hydrophobic (alkyl–sulfur)	5.06
ASP (A: 1040)	Hydrogen Bonding	2.57, 2.63

**Table 16:** A summary of types of interactions detected between propranolol and vitamin D3 receptors.

Amino Acid	Bond or Interaction	Distance (A°)
TYR (A: 143)	Hydrophobic (alkyl–cation)	4.92
	Pi-Sigma	3.66
LEU (A: 233)	Hydrophobic (alkyl–alkyl)	3.92, 4.75
ARG (A: 274)	Positive–Positive	4.50
SER (A: 278)	Hydrogen Bonding	2.79
TRP (A: 286)	Pi-Pi Stacking	4.67, 5.13

## Data Availability

The data presented in this study are available on request from the corresponding author.
